# Differential working memory function between phonological and visuospatial strategies: a magnetoencephalography study using a same visual task

**DOI:** 10.3389/fnhum.2023.1218437

**Published:** 2023-08-23

**Authors:** Hayate Onishi, Koichi Yokosawa

**Affiliations:** ^1^Graduate School of Health Sciences, Hokkaido University, Sapporo, Japan; ^2^Faculty of Health Sciences, Hokkaido University, Sapporo, Japan

**Keywords:** working memory, phonological strategy, visuospatial strategy, magnetoencephalography (MEG), executive functions

## Abstract

Previous studies have reported that, in working memory, the processing of visuospatial information and phonological information have different neural bases. However, in these studies, memory items were presented via different modalities. Therefore, the modality in which the memory items were presented and the strategy for memorizing them were not rigorously distinguished. In the present study, we explored the neural basis of two working memory strategies. Nineteen right-handed young adults memorized seven sequential directions presented visually in a task in which the memory strategy was either visuospatial or phonological (visuospatial/phonological condition). Source amplitudes of theta-band (5–7 Hz) rhythm were estimated from magnetoencephalography during the maintenance period and further analyzed using cluster-based permutation tests. Behavioral results revealed that the accuracy rates showed no significant differences between conditions, while the reaction time in the phonological condition was significantly longer than that in the visuospatial condition. Theta activity in the phonological condition was significantly greater than that in the visuospatial condition, and the cluster in spatio-temporal matrix with *p* < 5% difference extended to right prefrontal regions in the early maintenance period and right occipito-parietal regions in the late maintenance period. The theta activity results did not indicate strategy-specific neural bases but did reveal the dynamics of executive function required for phonological processing. The functions seemed to move from attention control and inhibition control in the prefrontal region to inhibition of irrelevant information in the occipito-parietal region.

## 1. Introduction

Working memory (WM) is responsible for the temporary maintenance and manipulation of information to carry out certain behavioral goals (Goldman-Rakic, 1995; Fuster and Bressler, 2012; Miller et al., 2018, as reviews), which is a higher cognitive function that is essential in daily life.

In the classical psychological model of WM (Baddeley and Hitch, 1974; Baddeley, 2012), WM comprises a phonological loop that stores phonological information, a visuospatial sketchpad that stores visuospatial information, and an executive function (central executive) that manipulates the information stored by these storage mechanisms. The neural basis of WM has been studied using a variety of tasks. For instance, studies using auditory stimuli or visual words have revealed brain activity in temporo-parietal and inferior frontal regions (Deng et al., 2011; Fegen et al., 2015; Albouy et al., 2019), while studies using objects or spatial positions revealed brain activity in occipital and parietal regions (Todd and Marois, 2004; Curtis, 2006; Sobczak-Edmans et al., 2016). These studies have typically adopted either phonological or visuospatial information, and have not always excluded the possibility that different subjects used different memory strategies to manipulate the information. In addition, because these studies were based on functional magnetic resonance imaging, the temporal variation of WM function has not been clarified. As described above, it remains unclear how WM function and its temporal variation differ when the same information is manipulated using different strategies.

Non-invasive electrophysiological methods [i.e., electroencephalography and magnetoencephalography (MEG)] are appropriate for recording temporal variation in brain activities. Various bands of brain rhythms have been studied to investigate memory processing (Klimesch, 1999; Crivelli-Decker et al., 2018, for review). Couplings between bands of brain rhythms have also been investigated, on the basis of Lisman and Idiart’s model (Lisman and Idiart, 1995). For example, Bahramisharif et al. (2018) investigated not only the theta/alpha-band (7–13 Hz) power during maintenance, but also the couplings between gamma-band (>30 Hz) power and the theta/alpha-band phase. The researchers concluded that the increase in theta/alpha-band power reflects the inhibition of upcoming sensory irrelevant information and the protection of the information that is already held in WM (Bahramisharif et al., 2018). Malenínská et al. (2021) reported that the theta to gamma cycle length ratio predicted memory performance using the digit span test. In contrast, within single bands of brain rhythms, an amplitude increase of the hippocampal theta-band (5–7 Hz) rhythm and an amplitude decrease of the cortical alpha-band (8–13 Hz) rhythm have been reported in learning and memory (Parish et al., 2018). Costers et al. (2020) demonstrated that theta activity reflects sensory processing in a study using the n-back task. Mizuhara et al. (2004) and Magosso et al. (2021) suggested that theta activity is associated with executive function. The alpha-band rhythm has advantageous characteristics for study, with a large amplitude and wide modulation, and there is considerable evidence indicating its involvement in short-term memory and WM processing (Foster et al., 2016; Wianda and Ross, 2019). Therefore, we compared the roles of alpha- and theta-band rhythm in our previous study (Takase et al., 2019). When young participants performed a sequential memory task similar to that used in the current study, the alpha-band rhythm contributed exclusively to the active inhibition of task-irrelevant inputs, whereas cortical theta-band rhythm was associated with memory performance. Thus, in the current study, we focused on the cortical theta-band rhythm.

In the current study, we recorded MEG while subjects performed a WM task that required two different memory strategies while presenting the same visual stimuli. We estimated the source amplitude of the theta-band rhythm during the period in which subjects maintained the information and rehearsed it in two different ways. By applying the cluster-based permutation test and effect sizes method (Meyer et al., 2021), we aimed to identify brain regions in which theta activities differed between strategies and how these differences varied over time. We expected to find a specific neural basis for each strategy.

Visually presented words are recognized by their shape, sound, and meaning, whereas objects placed in space are recognized by their shape, position, and angle. The recognition processes for visually presented words and objects involve the temporo-parietal and inferior frontal regions, and occipital and parietal regions, respectively. The previous studies mentioned above have generally reported effects that correspond to these brain areas (e.g., Sobczak-Edmans et al., 2016; Albouy et al., 2019). However, it remains unclear which brain areas are involved when the same visual stimuli are presented. Additionally, it is not known whether there is a difference in temporal variation between the different memory strategies during maintenance. When different processes are performed on the same visual stimuli, executive function is likely to be more important. Recent neuroscientific findings have suggested that maintenance and manipulation are accomplished by executive functions involving attention control, prioritizing information, inhibiting irrelevant information, and updating information (Diamond, 2013; D’Esposito and Postle, 2015; Myers et al., 2017; Chiu et al., 2018). Internal attention to representations is particularly important for maintenance (Banich et al., 2000; Magosso et al., 2021). Executive functions have been reported to involve prefrontal and parietal regions (Leung and Zhang, 2004; Mogadam et al., 2018; Schumacher et al., 2019). To detect the temporal dynamics of memory processing of different memory strategies, including executive function, we considered that it was necessary to take a spatial-temporal exploratory approach.

**FIGURE 1:**
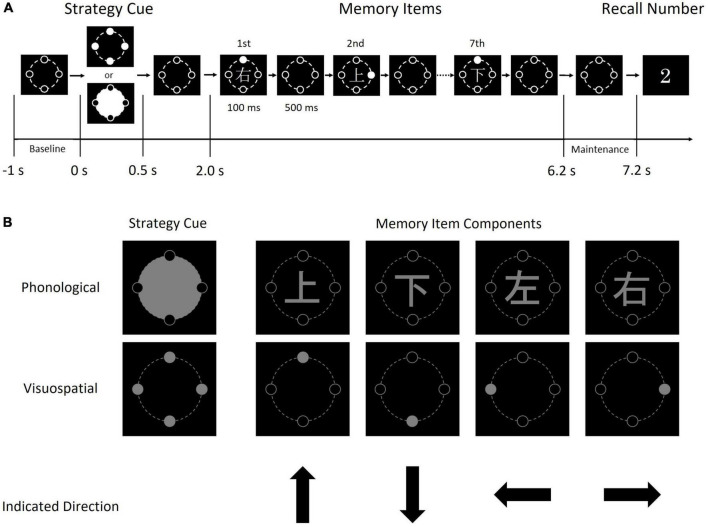
Schematic diagram of one trial of the sequential memory task **(A)** and the details of each image **(B)**. Each memory item consisted of a Kanji character and a gray circle. The condition cue indicates the condition (i.e., phonological or visuospatial); when the upper image (a large gray circle with four black circles) was presented, each subject was required to memorize the direction indicated by the Kanji character, by reading and memorizing it. In contrast, when the lower image (four gray circles) was presented, each subject was required to memorize the direction indicated by the position of the solid gray circle.

As a WM task, we designed a visual sequential memory task in which subjects memorized one of four (up, down left, right) directions. Subjects watched a white circle indicating the direction and a Kanji character indicating a direction at the same time and memorized one of them (Figure 1). Thus, subjects were forced to choose either a phonological or visuospatial strategy to maintain the information and perform rehearsal. Subjects reported the direction by pressing one of four directional buttons in our task, in contrast to traditional WM tasks in which subjects typically give two-choice (i.e., yes/no) responses. Thus, we were able to assess accuracy more rigorously using our WM task, because subjects were given four choices.

## 2. Materials and methods

### 2.1. Subjects

Nineteen right-handed young adults took part in the experiment [22.1 ± 1.1 years old (mean ± standard deviation), 12 males, seven females]. Subjects reported no history of neurological or psychiatric disorders and all reported normal or corrected-to-normal vision. The study was approved by the Ethics Committees of Faculty of Health Sciences at Hokkaido University. Written informed consent was obtained from each subject prior to the experiments.

### 2.2. Sequential memory task

Subjects were instructed to perform the visual sequential memory task shown in Figure 1, involving static images of a condition cue, memory items, and recall number. The task was created using Presentation (Neurobehavioral Systems, ver. 20.3, Berkeley, CA, USA), and memory items were presented in gray on a black background. The fonts of Kanji characters and recall numbers were Yu Gothic and Lucida Console, respectively. These images were presented using a back-projection screen. The screen was located in the magnetically shielded room for MEG recoding, while the projector was located outside.

First, a condition cue (i.e., phonological or visuospatial) was presented. Seven memory items consisting of Kanji characters (上, 下, 右, and 左 and mean up, down, right, and left, respectively) and a solid gray circle (positioned up, down, right, or left), were presented in order. Finally, a recall number from 1 to 7 was presented. In the phonological strategy, subjects were instructed to read and memorize a Kanji character. In the visuospatial strategy, subjects were instructed to memorize the positions of the solid gray circles. Thus, subjects memorized the directions using different strategies. Subjects answered the direction of the memory item at the serial position corresponding to the recall number by pressing one of four directional buttons with the right index finger. That is, if the recall number was 2 (see Figure 1), the subject was instructed to press the up button in the phonological condition because “上” represented “up,” and to press the right button in the visuospatial condition.

The condition cue was presented for 500 ms. After 1,500 ms from the disappearance of the condition cue, seven memory items were presented for 100 ms with a 600-ms interval. After 1,500 ms from the disappearance of the last memory item, the recall number was presented for 2,500 ms (“2” in Figure 1). The recall number vanished promptly after the subject pressed a button.

The experiment was composed of four task blocks for each subject. One task block included 56 trials with 28 phonological and 28 visuospatial conditions. Each recall number appeared four times in one block. Condition and recall number were presented pseudo-randomly in each task block.

The answered directions and the duration between the onset of the recall number and the moment of button pressing were recorded automatically to calculate the accuracy rates and reaction times. Here, 0 s denotes the onset of the condition cue. The time periods of −1 to 0 s and 6.2–7.2 s were classified as the baseline period and maintenance period, respectively. Inter-trial intervals were randomly selected between 3 s and 4 s.

### 2.3. MEG recording

Magnetoencephalography was recorded using a 101-channel helmet-shaped magnetometer system (customized; Elekta-Neuromag) installed at Hokkaido University, Sapporo, Japan. The passband was 0.1–100 Hz and the sampling rate was 600 Hz. The recordings were conducted in a magnetically shielded room. In each recording, the helmet-shaped sensor-array was tilted forward 10°. Three head position indicator coils were attached at subjects’ left and right pre-auricular points and nasion, and the head points were obtained using a digitizer according to standard MEG operating procedure (Takase et al., 2022). Each subject was seated in the MEG measurement chair, onto which a table was attached. On the table, a cross-key was placed at a comfortable position for pressing the button with the right hand. To prevent the subject from moving their head to look at the cross-key, the cross-key and right hand were covered with a box so that they were not visible to the subject. The distance between the screen and a subject was approximately 120 cm, and the diameter of the memory items was 7 cm. Therefore, the viewing angle was 3–4°. Each subject was instructed to keep looking straight ahead and not to perform eye movements.

### 2.4. Behavioral analyses

The accuracy rates and the reaction times were calculated according to each recall number. Two-way repeated analyses of variance (ANOVA), condition (phonological/visuospatial) × recall number (1–7), and *post hoc* analysis were performed on the accuracy rates and the reaction time separately. The significance threshold was set at 5%, and multiple comparisons were corrected using the Bonferroni method.

### 2.5. MEG preprocessing

Magnetoencephalography data were preprocessed and statistically analyzed using Brainstorm (Tadel et al., 2011), as follows. First, the MEG data from malfunctioning or excessively noisy sensors were removed. Next, mechanical noise or artifact caused by respirations or cardiac beats were eliminated using independent-components analysis. Noise characteristics of each MEG data were checked using Fourier transformation. MEG data recorded by sensors with prominent noise in the 1–40 Hz frequency range were removed. The remaining MEG data were band-pass filtered at 1–40 Hz. The filtered MEG data were extracted for each trial with −1 to 7.2 s (0 s denotes the onset of the condition cue). There were trials of 28 phonological and 28 visuospatial conditions in each of four task blocks. That is, there were 224 trials in total in one experiment. Extracted MEG data of each trial were checked by visual inspection. Data from trials with prominent artifacts were removed. To focus on amplitudes of theta-band (5–7 Hz) rhythm, the template evoked response was calculated and subtracted from the MEG data.

Current dipole moments at 15,002 vertices on the cortex were estimated by unconstrained minimum norm estimation. To estimate the shape of the individual brain, the head points of each subject were co-registered to the template brain and an overlapping-sphere forward model was computed prior to minimum norm estimation. Thus, the time series data of the three orthogonal components were obtained at 15,002 vertices on the estimated individual brain.

### 2.6. MEG analyses

The theta-band envelopes were computed in each orthogonal component using Hilbert transform. The L2 norm of the obtained envelope was then computed at each vertex. The results were averaged across all trials for each condition. The averaged envelopes were taken as the time series of theta activity in the following analysis. The theta activity data were transformed to standardized theta activity data by calculating the amplitude deviations, *X*_*std*_, which were the change rates of the amplitude against the mean amplitude within the baseline (−1 to 0 s) period. That is,

$$X_{s\,t\,d}=\frac{x-\mu }{\mu }\times 100,$$

where *x* is the amplitude at each time point, and μ is the mean amplitude within the baseline. 15,002 time series data points for standardized theta activities (%) were obtained for each subject and condition. To focus on memory maintenance, spatio-temporal data in the maintenance period (6.2–7.2 s) were extracted. The data were projected on the template brain to perform group statistics. To reduce the load on the computer, the data were down-sampled to 200 Hz (Costers et al., 2020).

### 2.7. Modulation of standardized theta activity

To survey the time course of the standardized theta activity overall, the standardized theta activity values for each condition were averaged over 15,002 vertices and all subjects.

### 2.8. Cluster-based permutation test

Spatio-temporal matrices (15,002 vertices × 200 time-points) of standardized theta activity values per subject of each condition were obtained via the process described above. To extract the difference in standardized theta activities between the phonological condition and the visuospatial condition from the spatio-temporal matrices, we adopted a cluster-based permutation test (Maris and Oostenveld, 2007) to reduce false positives caused by multiple comparisons. However, it has been noted that clusters do not provide precise spatial or temporal information (see Fieldtriptoolbox, 2020), because of the null hypothesis that both conditions come from the same distribution, that is, data were exchangeable. Nevertheless, the cluster provides the characteristics of the difference, which is informative when there is a lack of *a priori* information, as in this study.

Therefore, we selected brain regions of interest as follows. First, we calculated t-values of dependent samples *t*-test over the spatio-temporal matrices (15,002 × 200 samples) between two conditions and selected all samples whose t-value was larger than the threshold (the 97.5th quantile of a T-distribution). Next, we clustered the selected samples in connected sets based on spatial/temporal adjacency; the minimum number of samples was set as 2. We then calculated cluster-level statistics (SumT) by taking the sum of the t-values within each cluster. Finally, we took the largest SumT among the clusters.

In the next step, we performed a permutation test on the largest SumT. That is, we permutated the condition of the spatio-temporal matrices of each subject randomly and performed the above process to calculate the largest SumT. Although there were 2^19^ combinations, we performed 999 random combinations and constructed a histogram of the SumT. We confirmed that the largest SumT of the real combination was included in the largest 5% of the histogram (*n* = 1,000, alpha-level = 0.05). To ensure higher robustness, we narrowed down the cluster by extracting the samples with more than 100 adjacent vertices in each time point. The narrowed cluster was delineated at each time-point to determine regions of interest. That is, the brain regions of interest were decided by selecting the brain regions using Mindboggle atlas (Klein et al., 2005), which include the vertices in the narrowed cluster.

As we conducted the above process using a two-sided test, SumT could be bilateral: positive clusters with positive SumT values indicate that standardized theta activities of the cluster were larger for the phonological condition than those for visuospatial condition, whereas negative clusters with negative SumT indicate the opposite.

### 2.9. Effect sizes

It has been reported that a combination of the cluster-based permutation test and effect size evaluation (e.g., Cohen, 1988) is an effective approach for obtaining statistically robust results from large-scale data (Meyer et al., 2021). We therefore constructed spatio-temporal matrices of the standardized theta activities only for the brain regions of interest. Additionally, we calculated Cohen’s d of the samples (standardized theta activities) between conditions at each time point. We selected the time points including at least one vertex with a d-value above 0.8. A time span with a large effect (i.e., *d* > 0.8) of the condition on the theta activity was determined for each brain region of interest.

## 3. Results

### 3.1. Behavioral results

**FIGURE 2:**
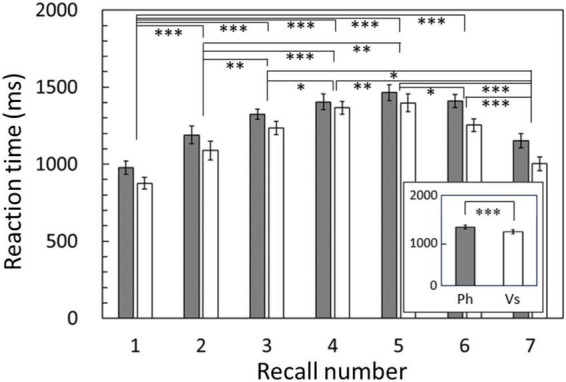
Reaction times averaged among subjects for each recall number and compared between conditions (inset). Reaction times in the phonological condition were significantly longer than those in the visuospatial condition (*p* < 0.001). Results of *post hoc* analysis for the recall number are also shown. Error bar: standard error. Ph or gray bars: phonological condition, Vs or white bars: visuospatial condition. *p < 0.05, **p < 0.01, ***p < 0.001.

**FIGURE 3:**
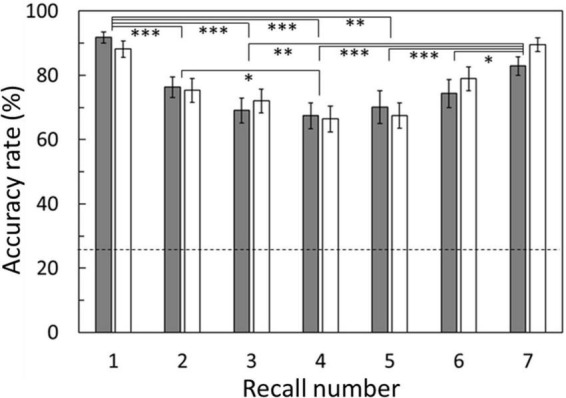
Accuracy rates averaged among subjects for each recall number. Accuracy rates exhibited no significant difference between conditions. Clear U-shaped serial position effects were shown in both conditions. Results of *post hoc* analysis for the recall number are also shown. Error bar: standard error. Gray bars: phonological condition, white bars: visuospatial condition. *p < 0.05, **p < 0.01, ***p < 0.001.

Regarding accuracy rates, two-way repeated ANOVA revealed a significant main effect of recall number [*F*_(2.834,51.021)_ = 13.517, *p* < 0.001]. There was no main effect of condition [*F*_(1,18)_ = 0.147, *p* = 0.706], and no interaction between condition and recall number [*F*_(4.164,74.961)_ = 1.214, *p* = 0.312]. Regarding reaction time, the results revealed significant main effects of both condition [*F*_(1,18)_ = 23.246, *p* < 0.001, Figure 2, inset] and recall number [*F*_(2.258,40.647)_ = 30.854, *p* < 0.001]. There was no interaction between condition and recall number [*F*_(3.538,63.688)_ = 1.113, *p* = 0.355]. For both accuracy rate and reaction time, the degrees of freedom were corrected using the Greenhouse–Geisser method. The *post hoc* analysis showed significant differences between recall numbers in both accuracy rate and reaction time (Figures 2, 3). That is, sequential position effects, a characteristic of sequential memory tasks, were clearly observed. We investigated the relationship between accuracy rate and brain rhythms focusing on the serial position effect adopting similar sequential memory tasks (Yokosawa et al., 2020). Nevertheless, this work aims to validate the differences between memory strategies (i.e., conditions). The main effect of the condition was observed only for reaction time, and there was no interaction with recall number. Therefore, in the subsequent discussion, we focused on the significantly longer reaction time in the phonological condition compared with that in the visuospatial condition.

### 3.2. Modulation of standardized theta activity

**FIGURE 4:**
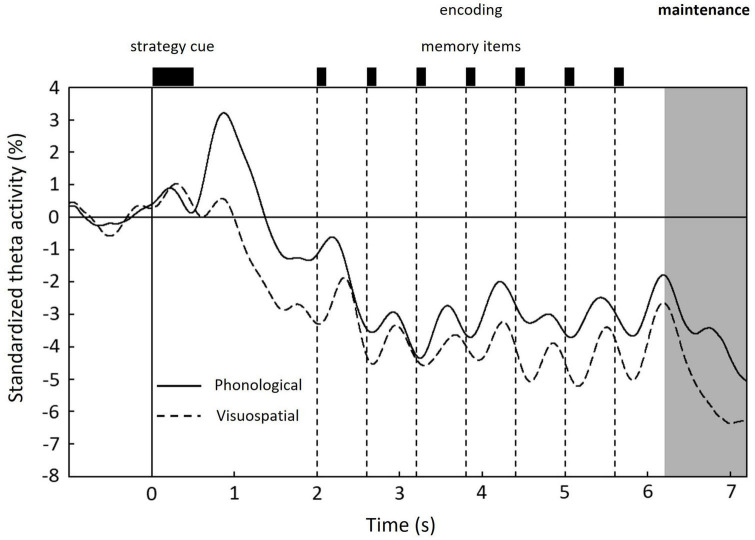
Time courses of standardized theta activity in the whole brain averaged over 15,002 vertices and grand-averaged among subjects for each condition: phonological condition (solid line) and visuospatial condition (dashed line). Unique peak during maintenance period is shown only in the phonological condition. 0 s denotes the onset of the strategy cue (see Figure 1).

The time courses of the standardized theta activity averaged over 15,002 vertices and grand-averaged among subjects are shown in Figure 4. In both conditions, the standardized theta activities peaked after the strategy cue presentation and decreased overall during encoding, exhibiting increases and decreases according to each memory item presentation. During the maintenance period, a distinctive peak was only observed in the phonological condition (indicated by an arrow in Figure 4).

### 3.3. Cluster-based permutation test

**FIGURE 5:**
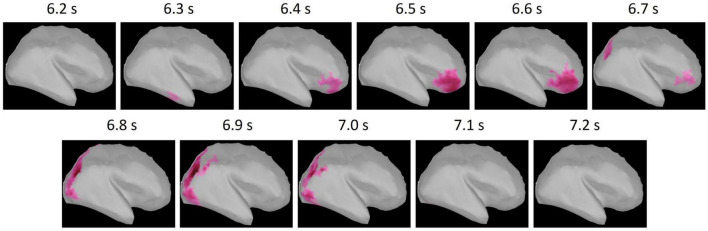
The positive (phonological condition >visuospatial condition) cluster obtained from cluster-based permutation test. The *t*-value of each vertex within the cluster is shown by the color maps. The cluster extended to right prefrontal regions in the early maintenance period (6.2–6.7 s) and the right occipito-parietal regions in the late maintenance period (6.8–7.2 s).

**FIGURE 6:**
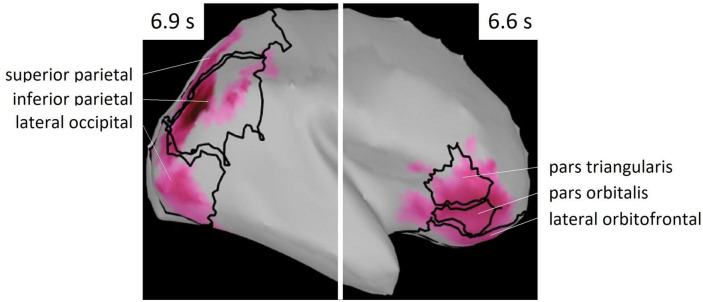
ROIs selected from Mindboggle atlas. These areas include vertices of the cluster shown in Figure 5.

Focusing on the distinctive difference between conditions observed in the maintenance period, we conducted a cluster-based permutation test on the standardized theta activities in the maintenance period. The results revealed that the standardized theta activity was significantly greater in the phonological condition compared with the visuospatial condition (*p* = 0.024). One two-dimensional positive cluster was observed in the spatial (15,002 vertices of the whole brain cortices) and temporal (200 time points in 1 s of the maintenance period) matrix with difference of the standardized theta activities between conditions (Figure 5). Thus, in the maintenance period, standardized theta activity values in the phonological condition were significantly larger than those in the visuospatial condition. The cluster extended to right prefrontal regions in the early maintenance period and right occipito-parietal regions in the late maintenance period. Given the particularly high reliability of MEG for signal sources in cortices that are close to the scalp, we extracted regions of interest (ROIs) on the lateral surface of the cortices (Figure 6).

### 3.4. Effect sizes

**FIGURE 7:**
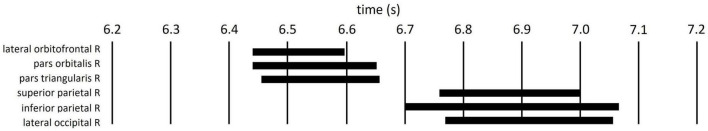
Time periods in which the ROI included the vertices with effect sizes greater than 0.8 (black bars).

**FIGURE 8:**
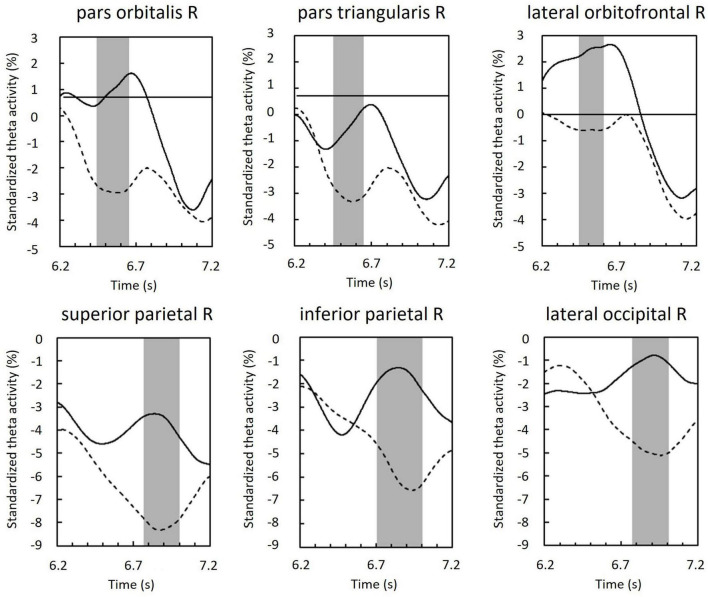
Standardized theta activity in the maintenance period of ROIs; those of the phonological condition (solid line) and visuospatial condition (dashed line). Gray shadows show time periods in which the ROI included vertices with effect sizes greater than 0.8 (see also Figure 7).

Regions of interest were defined as brain regions that included the vertices, including in the cluster. Right lateral orbitofrontal, pars orbitalis, pars triangularis, superior parietal, inferior parietal, and lateral occipital regions were selected on the basis of Mindboggle atlas (Figure 6). In each ROI, effect sizes were calculated at each time-point and vertex. We considered that the effect size was sufficiently large (i.e., valid) when the value of d was greater than 0.8, in accord with a previous study (Cohen, 1988). Figure 7 summarizes the time periods in which the effect sizes were greater than 0.8. Figure 8 shows the time traces of the standardized theta activities. The time periods in which each ROI included valid vertices are denoted by gray shadow.

## 4. Discussion

Behavioral data revealed that the reaction times were significantly longer for the phonological condition than those for the visuospatial condition, although the accuracy rates did not significantly differ between conditions (Figure 2). MEG data revealed that theta activity was significantly larger for the phonological condition than that for the visuospatial condition (Figure 5). Cluster-based permutation test and effect size data revealed that the difference was large in the prefrontal region in the early maintenance period, whereas it was large in the parietal and occipital regions in the late maintenance period (Figures 6, 7). Theta activity in the prefrontal and parietal regions is considered to reflect executive function in WM, especially that in the brain areas involved in attention control and task setting (Kawasaki et al., 2010; Sobczak-Edmans et al., 2016; Myers et al., 2017). The longer reaction time and greater theta activity associated with executive function suggests that a higher level of cognitive effort is involved in the phonological condition. Additionally, theta activity was larger in the phonological condition in the occipital region, which is considered to function as a visual association area. Positron emission tomography and functional magnetic resonance imaging studies have reported that activity in the occipital region is associated with maintenance of spatial WM (see Wager and Smith, 2003, for a review). In this study, we interpreted the theta activity in sensory areas as supporting the inhibition of irrelevant information (Bahramisharif et al., 2018; Kang et al., 2020). No brain regions were observed in which theta activity was larger in the visuospatial condition.

Below, we discuss the temporal variation in theta activity in each brain region (Figure 8). Here, the upward convex temporal variations indicate the transient of the theta-band rhythm. We assumed that these variations represent brain activity. The use of MEG allowed us to discuss such transient temporal variation.

### 4.1. Prefrontal theta

In the right inferior frontal region and orbitofrontal region, theta activity was larger in the phonological condition compared with that in the visuospatial condition in the early maintenance period (Figure 8). However, peaks (i.e., upward convex variations) in these areas were also observed in both visuospatial and phonological conditions, but were not found in the parietal or occipital regions. Considering that no theta activity specific to each condition was observed in any other brain regions, this may reflect the neural basis of strategy-independent executive function (Allen et al., 2012; Brown et al., 2012). This common peak may reflect rehearsal, particularly attention control, which is known to be important among the executive functions involved in rehearsal (Korsten et al., 2006). Below, we discuss the differentiated theta activity between conditions, referring to the functions of the subdomains of the prefrontal region, the inferior frontal gyrus, and the orbitofrontal cortex.

The ventrolateral prefrontal cortex, including the inferior frontal regions, is responsible for controlling and sustaining internal attention and applying top-down bias (task set) to information (Katsuki and Constantinidis, 2014; Weintraub-Brevda and Chua, 2019). In the phonological condition, reading, i.e., the sound of the word corresponding to the character, must have been preferentially stored in WM storage by applying bias to reading among the multiple properties of the Kanji character, such as shape, reading, and meaning. Thus, this function is already performing attentional selection (external attention) during encoding. During the maintenance period, subjects were asked to covertly rehearse the reading of the Kanji characters in the phonological condition. However, each Kanji character has not only reading, but also shape and meaning information. Thus, it is possible that other types of information were automatically recalled upon rehearsal (Pattamadilok et al., 2017). Some previous findings suggest that the meaning of a word is recalled more automatically than the color or shape of the word (Cohen et al., 1990). Our results indicate that the task set and sustaining internal attention were more strongly required in the phonological condition compared with those in the visuospatial condition (Zhang et al., 2003). In a study of orbitofrontal-injured patients, the patients were not impaired on tests of simple maintenance, but only on tests that required maintenance, monitoring, or manipulation (Barbey et al., 2011). It has also been suggested that the orbitofrontal region is involved in decision-making and manipulation of representations for goal-directed behavior (Kringelbach, 2005 as review). Our results suggest that the monitoring of mental representations is important for efficient rehearsal.

These results suggest that inferior frontal and orbitofrontal regions are involved in attention control for rehearsal that is common to both strategies. In addition, the results suggest that a high level of effort is required for execution to memorize Kanji characters that indicate directions, compared with that for memorizing directions visuospatially.

### 4.2. Parietal theta

In the right superior and inferior parietal regions, theta activity was greater in the phonological condition compared with that in the visuospatial condition in the late maintenance period (Figure 8). The superior parietal region is associated with the manipulation and rearrangement of information. For example, a study of patients with damage to the superior parietal lobule reported that this region was not involved in mere maintenance or recall of information, but was only involved in manipulation and rearrangement of information (Koenigs et al., 2009). Our results suggest that more complex manipulation is required in the phonological condition compared with that required in the visuospatial condition.

In addition, parietal regions, especially the inferior parietal region, are considered to be involved in an attentional functional network together with the prefrontal cortex (Leung and Zhang, 2004; Corbetta and Shulman, 2011; Kuo et al., 2012). Banich et al. (2000) reported activity in prefrontal and parietal regions during the Stroop task. They concluded that the inferior parietal region modulates attention to information that is automatically recalled in conjunction with the properties prioritized by the task set received from the prefrontal cortex and corrects the gap, and reported that the parietal regions are sensitive to irrelevant information. In a study of cross-modal conflict, the inferior parietal regions, together with the frontal regions, were reported to play an important role in the inhibition of a distractor (Sawamura et al., 2022). The increase in theta activity in the parietal regions can be interpreted as playing a role in controlling the focus of attention, to avoid attending to spatial information, or to inhibit irrelevant information.

Although parietal regions have been reported to perform executive functions together with frontal regions, the present results suggest that parietal regions are active only under conditions in which there is information that interferes with the complex manipulation and rehearsal of mental representations. The present results may provide evidence that rehearsal is regulated by such an attentional functional network.

### 4.3. Occipital theta

In the right lateral occipital region, theta activity was greater in the phonological condition compared with that in the visuospatial condition in the late maintenance period (Figure 8). In a study by Kawasaki et al. (2010) comparing verbal and spatial WM tasks, phase synchronization was found between frontal theta reflecting executive function, and alpha-band rhythm reflecting modality-specific sensory processing (in verbal, left temporal; in spatial, parietal). The authors concluded that alpha activity in sensory areas reflects storage buffers (Kawasaki et al., 2010). In recent studies, during encoding, increases in theta activity in occipito-parietal regions have been commonly observed, and have been shown to reflect sensory processing of visual information and attention to visual stimuli (Dugué et al., 2015; Pisella, 2017; Takase et al., 2022).

It is unsurprising that the auditory cortex was not activated in the phonological condition in our work, because visual stimuli were presented in both the visuospatial and phonological conditions. However, unexpectedly, greater theta activity was observed in a vision-related brain region in the phonological condition compared with that observed in the visuospatial condition. Because the task in the present study required subjects to respond to direction, it may also reflect a gating-like function that permits storage of automatically recalled spatial representations. However, the longer reaction time in the phonological condition suggests that the spatial representations were recalled on the basis of stored phonological representations after the recall number was presented. Hence, this interpretation may be questionable. Rather, we speculate that theta activity in the phonological condition may reflect inhibition of irrelevant spatial information, which should be automatically recalled during maintenance, along with parietal regions.

## 5. Limitations

Previous studies have reported modality-specific brain activities in sensory areas using multi-modal WM tasks. We also expected to find a specific neural basis for each strategy, by controlling the strategies for the same visual WM task to memory directions. However, we were not able to differentiate strategy-specific brain activities. This may suggest that such a difference occurs in other memory stages (i.e., encoding or recall) or that a difference is observed in rhythms with other frequency bands. The difference may be observed not in a rhythm with a single frequency band, but in cross-frequency (e.g., gamma-theta) coupling as reported by Bahramisharif et al. (2018).

Another limitation involved in the current study is that the phonological condition was more complex to process. This is likely to have occurred because the identified brain regions were exclusively involved in the phonological condition. Improvements will be needed in future study designs to align the complexities between conditions, including having participants respond in a phonological context. Further studies will be necessary to further elucidate this issue in future.

## 6. Conclusion

We controlled the working memory strategies (visuospatial and phonological) used by subjects to memorize directions presented visually and sequentially. Source amplitudes of the theta-band (5–7 Hz) rhythm estimated using MEG during the maintenance period were analyzed using cluster-based permutation tests. Brain activity and temporal dynamics were compared between strategies. Theta activity reflecting executive functions (particularly attention control and inhibition control) was characteristically observed in the phonological condition, and these activities were mainly supported by temporal dynamics from the frontal to the parietal regions. Furthermore, the findings suggested that not only attention to relevant information but also inhibition of irrelevant information is important for enhancing information maintenance. This inhibition appeared to occur in parietal regions and sensory areas that represented the modality of irrelevant information.

## Data availability statement

The raw data supporting the conclusions of this article will be made available by the authors, without undue reservation.

## Ethics statement

The studies involving humans were approved by the Ethics Committees of Faculty of Health Sciences at Hokkaido University. The studies were conducted in accordance with the local legislation and institutional requirements. The participants provided their written informed consent to participate in this study.

## Author contributions

HO was the primary contributor for all aspects of this study, including experimental design and execution, data analysis, and writing the manuscript. KY contributed to experimental design, data analysis, editing and revising the manuscript, and approving the submitted version. Both authors contributed to the article and approved the submitted version.
